# 

**Published:** 1895-06-15

**Authors:** 


### CONTENTS.

Hospital Sunday in London - - - - - - - - - 177

A Word to Living Londoners - - - - - - - - 178

Cremation on the Battlefield - - - - - - - - 178

Chloroform Fatalities.—I. By Sir BENJAMIN WARD RICHARDSON - - - - - - - - - - - - - - - 179

Are Women Worse than Men? - - - - - - - - 180

Notes and News - - - - - - - - - - - 182

Medical Progress and Hospital Clinics—

Two Cases of Membranous, or Croupous, Laryngitis in Infants, Treated by Intra-Laryngeal Applications, with Recovery - 183

PROGRESS IN MEDICINE.—Diseases of Children - - - - 184

PROGRESS IN SURGERY.—Antiseptics - - - - - 185

NEW APPLIANCES AND THINGS MEDICAL - - - - - 186

Annotations.—Heart Troubles at Halifax—The General Medical Council and the Midwives Bill—Anti-toxin for Snake-bite - 181

The General Medical Council - - - - - - - 187

The Institutional Workshop:—

HOSPITAL ADMINISTRATION.—The Position of Charing Cross and King's College Hospitals - - - - - - - - - 189

THE STORY OF THE INSANE FROM YEAR TO YEAR - - - 190

PRACTICAL DEPARTMENTS.—Floors for Hospital Wards - - 191

Around the Hospitals - - - - - - - - - - 191

Editor's Letter Box - - - - - - - - - - - 192

Scraps and Gleanings - - - - - - - - - - 192

The Book World of Medicine and Science - - - - 193

The Student of Medicine - - - - - - - - - 193

EXTRA SUPPLEMENT.—THE NURSING MIRROR.

News from the Nursing World.—Medals for Nurses—Not Altogether Monotonous—Indulgence in Drugs—St. Saviour's Hospital—Novel Qualifications for Nursing—Nurses' Work, Past and Present—Nursing in Holland—Training in Finland —Nurses at San Francisco—The Training of Deaconesses in U.S.A.—New Work for Women—Short Items - - - lxxii

Dress and Uniforms—St. George's Hospital - - - - lxxv

Where to Go - - - - - - - - - - - - - lxxv

Elementary Anatomy and Surgery for Nurses. By W. McADAM ECCLES, M.D., M.S., F.R.C.S. XXI.—The Respiratory System - - - - - - - - - - - lxxiii

Macclesfield Infirmary - - - - - - - - - lxxiv

Our American Letter - - - - - - - - - - lxxiv

Notes and Queries - - - - - - - - - - - lxxiv

School for the Blind at York - - - - - - - lxxvi

Appointments—Minor Appointments - - - - - - lxxvi

